# Supplementary material for: The causal relationship between the human gut microbiota and pyogenic arthritis: a Mendelian randomization study
Source: Front Cell Infect Microbiol. 2024 Nov 26;14:1452480. doi: 10.3389/fcimb.2024.1452480 (PMC11629706; doi:10.3389/fcimb.2024.1452480)
Supplement: Supplementary file 1 [file DataSheet1.zip › mendelian test/ebi-a-GCST90027501.csv_leave_one_out.pdf]

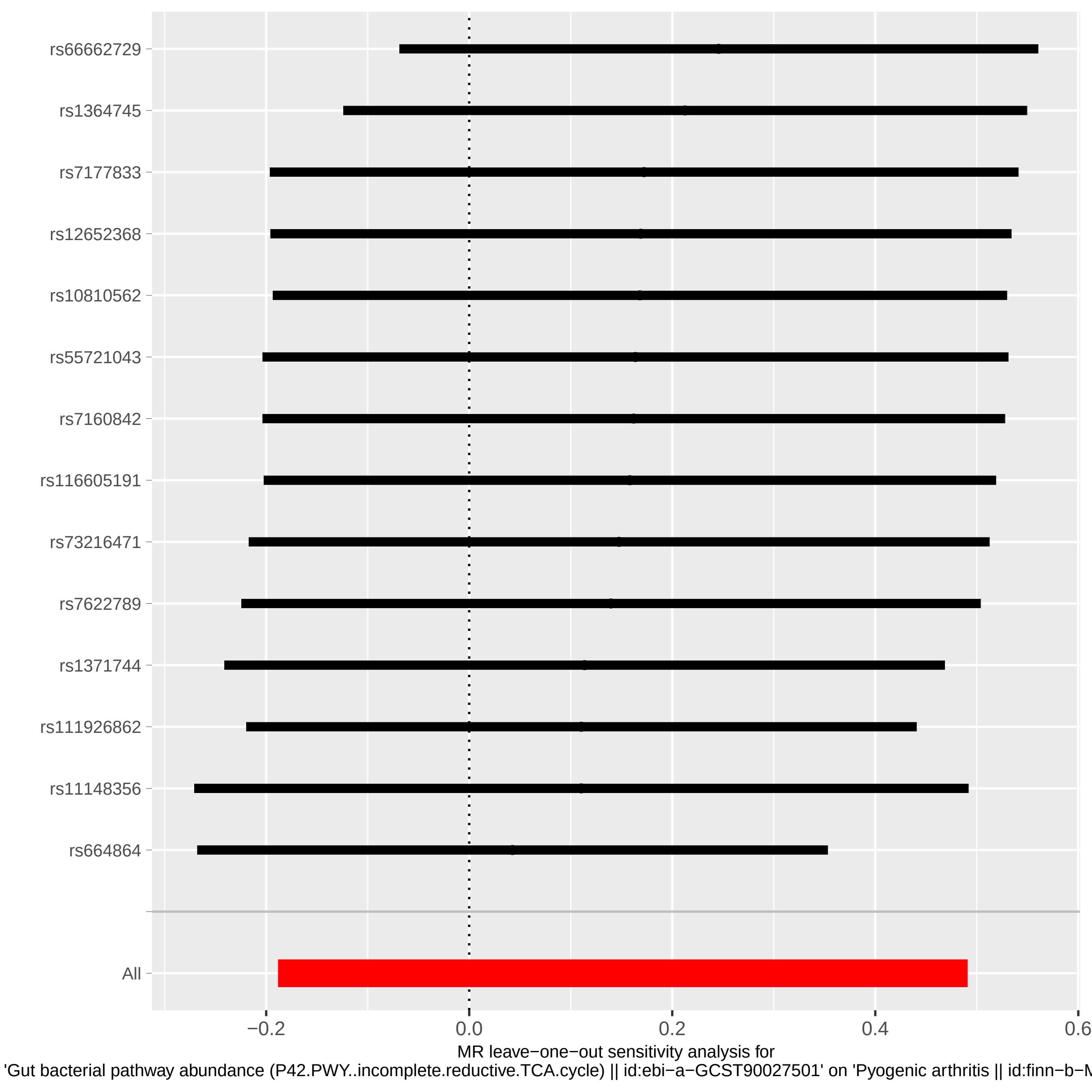
rs66662729
rs1364745
rs7177833
rs12652368
rs10810562
rs55721043
rs7160842
rs116605191
rs73216471
rs7622789
rs1371744
rs111926862
rs11148356
rs664864
All
−0.2
0.0
0.2
0.4
0.6
MR leave−one−out sensitivity analysis for
'Gut bacterial pathway abundance (P42.PWY..incomplete.reductive.TCA.cycle) || id:ebi−a−GCST90027501' on 'Pyogenic arthritis || id:finn−b−M
